# Association between serum high-sensitivity C-reactive protein levels and erectile dysfunction: a cross-sectional study of Chinese male population

**DOI:** 10.1038/s41598-019-42342-3

**Published:** 2019-04-11

**Authors:** Wenying Li, Kai Chen, Jinping Zhang, Xiaohong Wang, Guangyu Xu, Yinghong Zhu, Yan Lv

**Affiliations:** 1 Department of Obstetrics and Gynecology, Center for Reproductive Medicine, The People’s Hospital of LaiWu City, LaiWu Affiliated Hospital of Taishan Medical College, Shandong Province, China; 2 Center for Disease Control and Prevention of LaiWu City, Shandong Province, China; 3 Department of Urology, The People’s Hospital of LaiWu City, LaiWu Affiliated Hospital of Taishan Medical College, Shandong Province, China

## Abstract

Inflammation has been known to affect endothelial function and is involved in the progression of erectile dysfunction (ED). Thus, our present study was conducted to investigate the association between inflammatory marker high-sensitivity C-reactive protein (hs-CRP) and ED in a Chinese male population. A total of 1515 participants with anthropometric measurements, serum analyses and hs-CRP values available were included in our cross-sectional study. Data involving socioeconomic and lifestyle factors were also collected. ED was assessed by the 5-item International Index Erectile Function (IIEF-5), and hs-CRP levels were measured by the immunoturbidimetric assay. Logistic regression was applied to estimate the association between the serum hs-CRP and the risk of ED, and receiver operating characteristics (ROC) curve analysis was performed to identify the predictive value of hs-CRP. Serum hs-CRP levels were significantly higher in ED patients, and increased progressively with the incremental severity of ED (*P* < 0.001 for trend). In the multivariate-adjusted model, men in the highest quartile of hs-CRP level versus those in the lowest quartile had a 50% increased likelihood for ED (OR = 1.50; 95% CI = 1.08–2.08). When subjects were stratified by age, the risk of ED was more prominently in the middle-aged and elderly men. Based on the ROC analysis, serum hs-CRP has a poor diagnostic value for ED with an AUC of 0.58 (95% CI: 0.56–0.61) but has a good diagnostic performance for differentiating severe ED (AUC: 0.79; 95% CI: 0.77–0.81). Our study indicates that increased serum hs-CRP levels are associated with the severity of ED and an increased ED risk in a Chinese male population. These findings suggest that hs-CRP may be of value as an inflammatory marker for the assessment of ED risk and may play an important role in the etiology of ED.

## Introduction

Erectile dysfunction (ED), which is defined as the consistent inability to attain or maintain a sufficient erection for satisfying sexual intercourse^1^, has a marked prevalence and adversely affects the quality of life. Despite not a life-threatening condition, ED can be distressing because of its significant negative impact on both the physical and psychological health of male patients, as well as the life satisfaction of the sufferers and their partners^2^. The reported prevalence of ED varied from 2% to 90% depending on the age, race, assessment tool, the extent of comorbidities and geographical location of the population studied. With the rising of comorbid conditions associated with ED worldwide, it is estimated that the incidence of ED will dramatically increase and that by 2025, nearly 320 million men will be affected by ED^3^. Although the process of ED is complex and multi-factorial, current evidence suggest that the most common etiology of ED often involves endothelium damage due to endothelial dysfunction and atherosclerosis, which is an essential physiopathological link between ED and cardiovascular disease (CVD)^4^. In addition, accumulating evidence support the fact that inflammation affects endothelial function and participates pivotally in all stages of atherosclerosis, from lesion initiation to progression and destabilization^5,6^.

High-sensitivity C-reactive protein (hs-CRP) is a sensitive and stable biomarker of inflammation that strongly and independently predict future CVD, including myocardial infarction, ischemic stroke, and vascular death^7^. Subsequent large epidemiological studies conformed that even small increases in serum hs-CRP are associated with a higher risk of atherosclerosis and ischemic heart disease in apparently healthy subjects^8–10^. Meanwhile, a number of clinical and basic science research clearly demonstrated that ED and CVD share many similar features and risk factors, namely age, hypertension, dyslipidemia, smoking, obesity, and diabetes, and ED may be an early sign of atherosclerosis and CVD^11^. In this context, these findings raise the hypothesis that hs-CRP as a inflammatory marker is closely associated with ED.

Currently, epidemiologic investigations implicating the relationship between hs-CRP and ED were mainly conducted in western populations and diseased populations (e.g., individuals with obesity, type 2 diabetes or hypertension)^12–15^. Nevertheless, the aforementioned studies were somewhat less consistent and were hampered by several limitations, including the relatively small sample size and limited adjusted confounding factors; hence, the generalization of these studies is limited. Only few studies have evaluated the relation between hs-CRP and ED in Chinese^16^, yet this study was also limited almost exclusively to data from young men without overt CVD. Besides, serum levels of C-reactive protein (CRP) vary distinctly between ethnic/racial groups^17^, and Chinese population seem to have relatively low levels of CRP^18^. Importantly, the prevalence of ED is increasing in China because of the rapidly aging populations and changes of the lifestyle during the past decades^19^. Hence, ED has become an important public health concern, and identifying and understanding the physiologically interrelated risk factors of ED have significance in promoting the appropriate preventative strategies and health services. Therefore, we sought to systematically and objectively evaluate serum hs-CRP as a risk marker of ED and to assess its predictive value in a large sample of Chinese males.

## Material and Methods

### Study Population

The participants were from the The Laiwu City Health Survey (LCHS), which is a population-based prospective cohort study aiming to investigate the reproductive and sexual health status in adults. From September 2014 to June 2015, the inception cohort was performed among the adults who took their health checks at the physical examination center of The People’s Hospital of LaiWu City, the largest and most comprehensive physical examination center in LaiWu, comprising standardized questionnaire, clinical examinations, and detailed laboratory assessment. All studied individuals who voluntarily participated in this study completed a comprehensive demographic characteristics survey and were recruited continuously.

**Figure 1:**
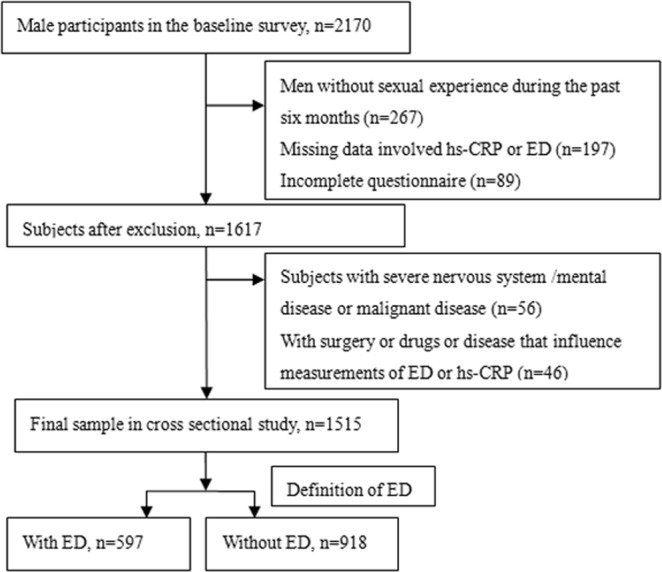
Flow chart for selection of study participants.

Briefly, LCHS, an ongoing large-scaled study, initiated from 2014 and followed up every 3–4 years, a total of 4267 subjects (2170 males and 2097 females, aged 18 years and over) completed the baseline survey. Written informed consent was obtained from all participants; our study was conducted according to the 2013 Declaration of Helsinki (revision) and was approved by the Ethics Committee of the People’s Hospital of LaiWu City. This study was reported according to the strengthening the reporting of observational studies in epidemiology (STROBE) guidelines^20^. The baseline data during 2014–2015 was used to perform a cross-sectional analysis in this study. In addition, participants with confounding factors influenced the assessment of ED or hs-CRP levels were excluded: (a) with incomplete questionnaire or laboratory data; (b) used medications that influence the genitourinary system, such as a-blockers or 5a-reductase inhibitors; (c) with pelvic surgery or trauma, prostatic disease; (d) currently having specific or nonspecific inflammation or taking antibiotic medications or/and hs-CRP value >10 mg/L; and (e) with nervous system /mental disease or autoimmune disease or malignant disease (e.g. myocardial infarction, congestive heart failure, hepatic failure, renal failure and malignant tumor). Ultimately, a total of 1515 participants were eligible and included in the present study. The detail screening process of subjects is shown in Fig. 1.

Standard and structured questionnaires were administered by well-trained interviewers via face-to-face interviews to collect information on demographics, history of disease (including common chronic diseases, malignant disease, mental disease, and so on.), medical history, health status, and lifestyle. Following the interview, the participant underwent a systemic physical examination. According to self-reported information, smoking status was classified as never, current and former. Participants who had regular activities for more than 30 minutes at least 3 times a week were considered physical activity, and those who had been drinking alcoholic beverages once or more weekly for at least half a year were considered current drinkers.

### Data collection and measurements

The anthropometric measurements were conducted by trained nurses using a standardized protocol. Height and weight were measured with barefoot to the nearest 0.1 cm and 0.1 kg, respectively. Body mass index (BMI) was calculated as the ratio between weight in kilograms and the height in meters squared (kg/m^2^). Being overweight was defined as BMI ≥24 and <28 kg/m^2^ and BMI ≥28 kg/m^2^ was considered as Obesity^21^. The blood pressure (BP) was measured repeatedly and the average values were taken. Hypertension was defined as having a history of hypertension, or taking anti-hypertensive treatment, or a systolic blood pressure (SBP) ≥140 mmHg, and/or a diastolic blood pressure (DBP) ≥90 mmHg. Similarly, Diabetes was defined as a history of diabetes, currently treated with oral hypoglycemic agents or insulin, or fasting blood glucose (FBG) ≥7.0 mmol/L^22^. Dyslipidemia was defined by serum levels of total cholesterol (TC) >6.22 mmol/L and/or Triglycerides (TG) >2.26 mmol/L and/or low-density lipoprotein cholesterol (LDL-C) >4.14 mmol/L and/or high-density lipoprotein cholesterol (HDL-C) <1.04 mmol/L, and/or a self-reported history and taking lipid-lower therapy according to Chinese guideline^23^.

### Laboratory measurements

Briefly, overnight fasting venous blood samples were collected from all participants and were separated immediately, some of which were stored at −80 °C until further analysis. Serum hs-CRP levels were measured by the immunoturbidimetric assay (TBA-200FR, Tokyo, Japan). Serum concentrations of total testosterone (TT) were measured using chemiluminescent immunoassays on COBAS system E602 immunoassay analyzer (Roche Cobas E602, Switzerland). The coefficients of variation for intra-assay and inter-assay variations were <2.0% for all analysis. All biochemical parameters including TG, HDL-C, LDL-C, TC and FBG were measured immediately and detected enzymatically on a Dimensional-RxL Chemistry Analyzer (Dade Behring, Newark, DE).

### Definition of ED

The participants who had a stable sexual relationship during the past 6 months and at least one sexual attempts within the last 1 month were used the 5-item International Index of Erectile Function (IIEF-5) questionnaire to assess diagnosis and severity of ED. The IIEF-5 scores consist of five questions and each item is scored on a 5-point scale with the lower results representing poorer sexual function^24^. Based on the diagnostic criteria of the ED, men with IIEF-5 score ranging from 22–25 were considered having normal erectile function, and the severity of ED was classified as mild (score 17–21), mild to moderate (score 12–16), moderate (score 8–11), and severe (score 5–7).

### Statistical Analysis

Variables collected at baseline were analyzed. The Kolmogorov-Smirnov test was conducted to test the normality. Continuous variables were expressed as mean ± SD or median (interquartile range), as appropriate. Differences of normal variables were assessed by the Student’s t-test and non-normal variables were compared by the Wilcoxon rank-sum (Mann-Whitney U) test. Comparisons of categorical variables were performed with chi-square test. The association of hs-CRP levels across different strata of ED was compared using ANOVA followed by trend analysis. Serum hs-CRP levels were categorized into 4 groups by quartiles^25^. Afterward, three logistic regression models were performed to estimate the association between hs-CRP and risk of ED, using the odds ratios (ORs) and 95% confidence intervals (CIs) and adjusting for potential confounding variables including age, BMI, TT, smoking, alcohol use, physical activity, diabetes, hypertension and dyslipidemia. In addition, receiver operating characteristics (ROC) curve was used to calculate the area under the curve (AUC), evaluating the diagnostic value of hs-CRP. The AUCs and optimal points were determined using MedCalc version 11.4.2.0 for Windows (MedCalc Software, Mariakerke, Belgium). Statistical analyses except ROC analysis were conducted with SPSS, version 13.0 (IBM Corp., Chicago, USA), and a *P*-value < 0.05 (two-tailed) was considered to indicate statistical significance.

## Results

The baseline characteristics of the study population stratified by ED are presented in Table 1. A total of 597 and 918 subjects diagnosed with ED and without ED (non- ED) were identified, and the overall prevalence of ED was 39.4% (597/1515). Subjects with ED were significantly older than men in the non-ED group (P < 0.001), yet there were no significant differences regarding BMI, TG and HDL-C between the subjects with ED and the control (non-ED) group. Although all the values were within the normal range, the levels of FBG, BP, TC, LDL-C and hs-CRP were significantly higher in subjects with ED compared to the control group, whereas the testosterone level was lower in the ED group. However, the proportion of smoking status, alcohol use, and physical activity did not show a significant difference between the two groups (all *P* > 0.05).

**Table 1:** Baseline characteristics in subjects according to presence of erectile dysfunction.

Variable^*^	Erectile dysfunction (n = 597)	No erectile dysfunction (n = 918)	*P*- value^†^
Age (y)	38 (31, 46)	34 (28, 40)	<0.001
IIEF-5	17 (14, 19)	23 (22, 24)	<0.001
SBP (mm Hg)	120 (110, 130)	118 (110, 126)	0.004
DBP (mm Hg)	80 (70, 84)	78 (70, 80)	0.024
BMI (kg/m^2^)	23.45 (20.98, 25.73)	23.03 (20.79, 25.27)	0.098
FBG (mmol/L)	5.47 ± 1.41	5.31 ± 1.21	<0.001
TG (mmol/L)	1.18 (0.80, 1.90)	1.17 (0.78, 1.74)	0.292
HDL-C (mmol/L)	1.35 (1.17, 1.57)	1.35 (1.18, 1.58)	0.945
LDL-C (mmol/L)	3.0 (2.52, 3.52)	2.88 (2.41, 3.40)	0.004
TC (mmol/L)	5.74 (5.06, 6.49)	5.53 (4.94, 6.19)	<0.001
TT (ng/ml)	6.07 ± 1.96	6.29 ± 1.90	0.030
hs-CRP (mg/L)	0.66 (0.31, 1.53)	0.48 (0.21, 1.03)	<0.001
**Smoking status**, **n** (**%**)			
Never	251 (42.1)	428 (46.6)	0.08
Former	27 (4.5)	30 (3.3)	0.21
Current	319 (53.4)	460 (50.1)	0.206
Alcohol consumption, (%)	508 (85.1)	782 (85.2)	0.96
Physical activity, (%)	209 (35.0)	346 (37.7)	0.29

^*^Median (interquartile range) or percentage.

^†^Differences between means were compared using unpaired Student’s t test or Mann-Whitney U test; Categorical variables were compared by χ^2^ test.

Abbreviations: SBP, systolic blood pressure; DBP, diastolic blood pressure; BMI, Body mass index;WC, waist circumference; FBG; fasting blood glucose; TG, triglyceride; TC, total cholesterol;HDL-C, high-density lipoprotein cholesterol; LDL-C, low-density lipoprotein cholesterol; TT, total testosterone; hs-CRP, High-sensitivity C-reactive protein.

**Figure 2:**
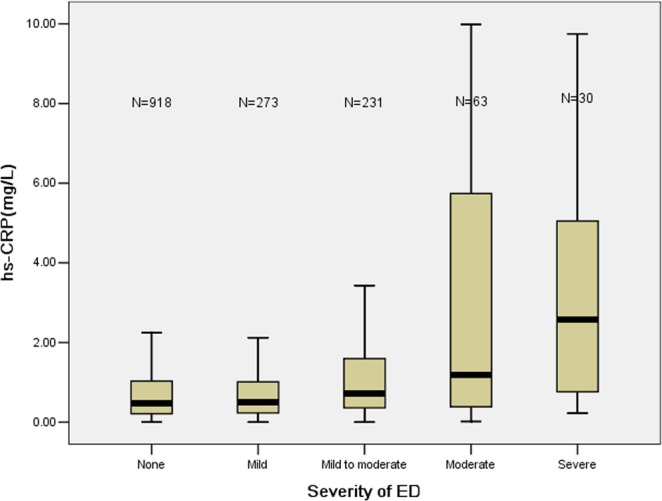
The serum levels of hs-CRP across different severities of ED groups.

In addition, serum levels of hs-CRP across different severities of ED groups are shown in Fig. 2. The study populations were stratified into five subgroups according to the severity of ED, and the median hs-CRP level in each group was 0.48, 0.50, 0.72, 1.19 and 2.57 mg/L, respectively. Notably, serum levels of hs-CRP were gradually elevated in parallel with the increased severity of ED (*P* < 0.001 for trend).

The logistic regression analysis was performed to illustrate the relationship between the elevated hs-CRP levels and risk of ED. Serum levels of hs-CRP were divided into quartile and the bottom quartile group was used as the reference. Three models were presented in the logistic regression analysis: the unadjusted, age-adjusted, and multivariate-adjusted, respectively. The logistic regression analysis and age-stratified analyses results are listed in Table 2. In the unadjusted analyses, compared with the participants in the 1st hs-CRP quartile, those in the 2nd, 3rd and 4th hs-CRP quartile had an OR of 1.19, 1.47 and 1.80 for ED, respectively. After adjustment for age, only the upper quartile of hs-CRP group still had a statistically significant relationship (OR = 1.51; 95% CI = 1.12–2.05), and the significant associations in the other two groups disappeared. Furthermore, the effect (OR = 1.50; 95% CI = 1.08–2.08) was slightly changed but remained significant after multivariate adjustment in the upper quartile of hs-CRP group. In addition, the risk of ED all increased progressively across the hs-CRP quartiles in three models (all *p* < 0.01 for trend). Because advancing age is a strong factor influencing ED, age-stratified analyses was conducted. The participants were classified as young (aged 20–39) group and middle-aged and elderly (aged 40–59 and ≥60) group, middle-aged and elderly males had significantly higher hs-CRP than young males (median 0.68 mg/l Vs 0.48 mg/l, *p* < 0.01). Once again, significant effects were found in the two subgroups when compared the top quartile to the bottom quartile of hs-CRP, especially in the middle-aged and elderly group (OR = 2.02; 95% CI = 1.18–3.47).

**Table 2:** Logistic regression analysis and age-stratified analyses results for the association between serum hs-CRP levels and ED.

Variable	Number	Unadjusted		Age-adjusted		Multivariate-adjusted*	
ALL participants	(n = 1515)	OR (95% CI)	*P*-value	OR (95% CI)	*P*-value	OR (95% CI)	*P*-value
**Quartile of hs-CRP**							
Q1 (<0.24 mg/L)	377	1	1	1	1	1	1
Q2 (0.24–0.53 mg/L)	372	1.19 (0.88, 1.61)	0.258	1.10 (0.80, 1.49)	0.563	1.08 (0.79, 1.48)	0.62
Q3 (0.54–1.22 mg/L)	380	1.47 (1.09, 1.98)	0.011	1.20 (0.88, 1.63)	0.257	1.19 (0.87, 1.64)	0.283
Q4 (>1.22 mg/L)	386	1.80 (1.35, 2.42)	<0.001	1.51 (1.12, 2.05)	0.008	1.50 (1.08, 2.08)	0.015
*P*-value for trend			<0.001		<0.001		0.003
Young group	(n = 985)						
Q1 (<0.20 mg/L)	259	1	1	1	1	1	1
Q2 (0.21–0.47 mg/L)	243	1.18 (0.80, 1.74)	0.395	1.15 (0.78, 1.69)	0.44	1.18 (0.80, 1.75)	0.406
Q3 (0.48–1.16 mg/L)	246	1.40 (0.96, 2.04)	0.083	1.34 (0.91, 1.96)	0.137	1.41 (0.94, 2.10)	0.093
Q4 (>1.16 mg/L)	237	1.62 (1.11, 2.37)	0.012	1.56 (1.07, 2.28)	0.022	1.72 (1.13, 2.59)	0.011
Middle aged and elderly group	(n = 530)						
Q1 (<0.32 mg/L)	132	1	1	1	1	1	1
Q2 (0.33–0.67 mg/L)	133	1.22 (0.75, 1.98)	0.42	1.21 (0.74, 1.99)	0.441	1.13 (0.68, 1.89)	0.628
Q3 (0.68–1.42 mg/L)	133	1.42 (0.87, 2.30)	0.157	1.30 (0.79, 2.13)	0.307	1.17 (0.70, 1.97)	0.553
Q4 (>1.42 mg/L)	132	2.46 (1.50, 4.04)	<0.001	2.31 (1.39, 3.84)	0.001	2.02 (1.18, 3.47)	0.011
*P*-value for trend			<0.001		<0.001		<0.001

^*^Logistic regression models were performed to estimate the odds ratios (ORs) and 95% confidence intervals (CIs); *P* values were calculated by logistic-regression analyses and adjusting for age, BMI, testosterone, smoking, alcohol use, physical activity, diabetes, hypertension and dyslipidemia in the multivariate-adjusted model.

**Figure 3:**
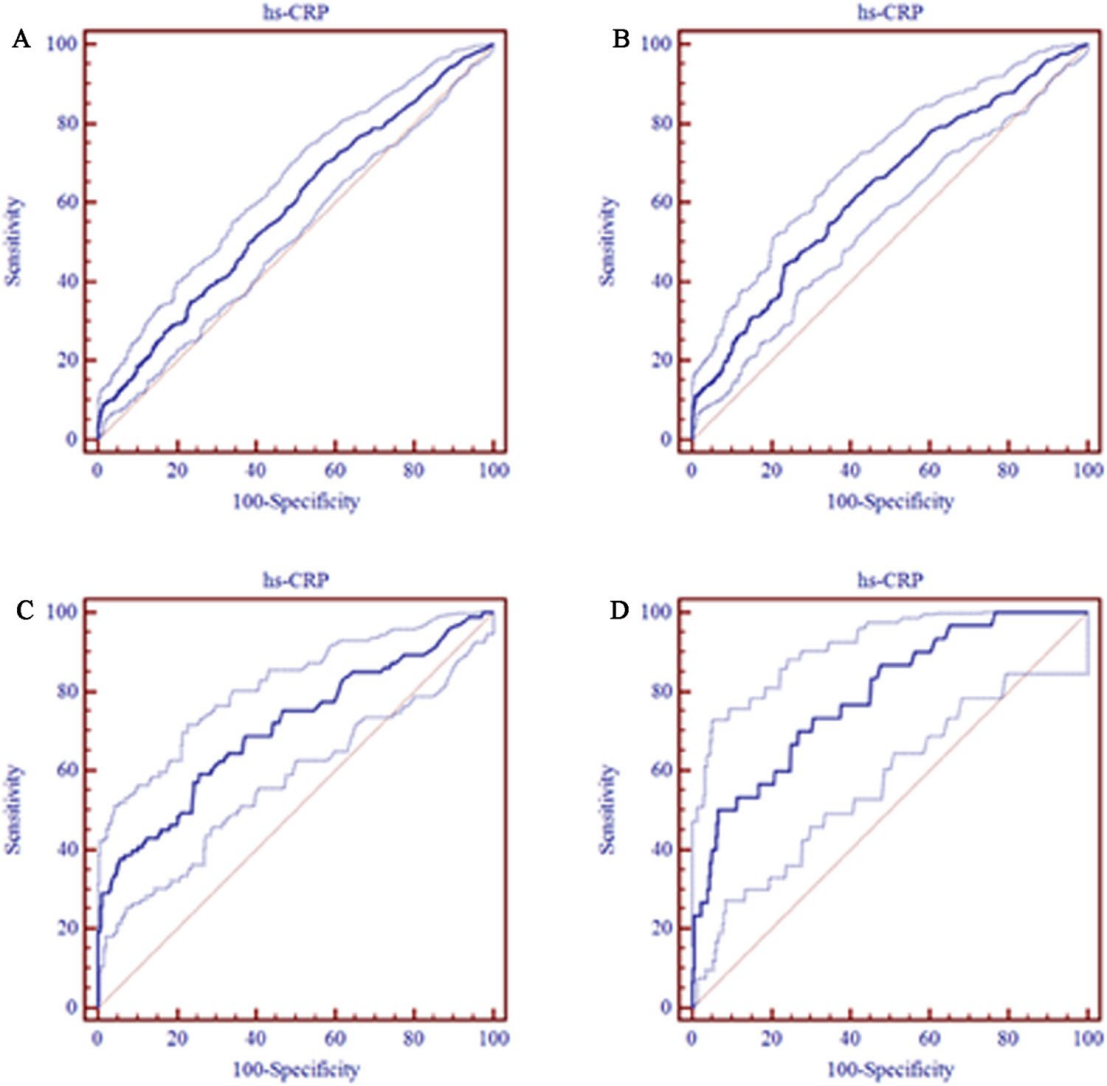
ROC curves analysis show the results of serum hs-CRP prediction in different severity of ED subgroups (including ROC curve graph with 95% Confidence bounds). (**A**) ROC curve for differentiating ED patients. AUC was 0.58 (95% CI: 0.56–0.61), and the cutoff value, sensitivity, and specificity were 0.37 mg/L, 70.2%, and 42.2%, respectively. (**B**) ROC curve for differentiating mild to severe ED. AUC was 0.63 (95% CI: 0.61–0.66), and the cutoff value, sensitivity, and specificity were 1.09 mg/L, 44.4%, and 76.3%, respectively. (**C**) ROC curve for differentiating moderate to severe ED. AUC was 0.71 (95% CI: 0.68–0.73), and the cutoff value, sensitivity, and specificity were 1.11 mg/L, 59.1%, and 74.2%, respectively. (**D**) ROC curve for differentiating severe ED. AUC was 0.79 (95% CI: 0.77–0.81), and the cutoff value, sensitivity, and specificity were 2.98 mg/L, 50.0%, and 93.3%, respectively.

ROC analysis was performed to evaluate the value of serum hs-CRP to predict different severities of ED, and the results are shown in Fig. 3. In addition to the degree of ED (score ≤21), we modified slightly by combining group into 1 of 3 total categories: 1) mild to severe ED (score ≤16), 2) moderate to severe ED (score ≤11) and 3) severe ED (score ≤7). Based on the ROC curve, serum hs-CRP has a poor diagnostic value for ED with an AUC of 0.58 (95% CI: 0.56–0.61). Among the three categories of ED, serum hs-CRP had the best diagnostic performance for severe ED. The optimal cut-off value of hs-CRP >2.98 mg/L had a sensitivity of 50.0% and a specificity of 93.3% (AUC = 0.79; 95% CI: 0.77–0.81) for predicting severe ED.

## Discussion

In this cross-sectional study, we estimated the association between hs-CRP levels and ED and assessed the diagnostic value of hs-CRP level. As expected, we observed that elevated levels of hs-CRP were significantly associated with an increased risk of ED after adjustment for conventional ED risk factors, including age, BMI, testosterone, smoking, alcohol consumption, physical activity, diabetes, hypertension and dyslipidemia. More importantly, our study highlighted the important relationship between hs-CRP levels and ED in a relatively large Chinese male population, extrapolating the result to a much broader population. Thus, our findings combined with previous data reinforce the current scientific evidence regarding the connection between hs-CRP and ED.

Currently, our data showed that serum hs-CRP levels were significantly higher among men with ED than in controls. This is in line with previous surveys, which reported that serum CRP levels were frequently high in men with ED in the obese or metabolic syndrome population^12,26^. Interestingly, a previous report showed that the hs-CRP levels in patients with ED only (ED/no-CVD) were even equivalent to that in patients with CVD only^27^. Furthermore, our finding that serum hs-CRP levels were elevated with increasing ED severity is similar to a previous case-control study^28^, in which a negative linear correlation between IIEF score and CRP levels was observed, suggesting that chronic low-grade inflammation as expressed by increased hs-CRP levels correlated with the severity of ED.

Meanwhile, our study indicated that increased hs-CRP levels are associated with a greater risk of ED and the increased risk of ED was more prominently in the middle-aged and elderly men on the basis of age-stratified analyses. In consistent with the published studies, we also found that middle-aged and elderly men had higher levels of serum hs-CRP than young men, which may be attributable to the differences in age, obesity, BP, glucose, comorbid conditions, lifestyle, and dietary habits^18,29,30^. It implied that inflammatory processes may develop with the advancing age and contribute more significantly to ED among older males to some extent. Several, but not all, epidemiologic studies have revealed a statistically significant association between hs-CRP and ED. For instance, Blans *et al*.^13^ showed that in men with diabetes, the risk of ED was significantly higher in the top tertile of hs-CRP concentration as compared with that in the bottom tertile (OR = 4.3). In another study^16^ focusing on young male with low cardiovascular risk, Yao *et al*. found that high hs-CRP levels were associated with a 2.8-fold increase in ED risk. Similarly, in ED patients with or without coronary artery disease (CAD), Vlachopoulos *et al*.^27^ documented that an increase in serum hs-CRP of 1 mg/L are associated with an approximately 2-fold increase in the risk of ED (95% CI; 1.18–2.74). These findings, however, have several weaknesses, such as the small sample size, selection bias, restricted participants (only for specific population groups or particular diseases), as well as the limited adjusted confounding factors. By contrast, in a retrospective trial, Eaton *et al*.^15^ found no significant association between ED and CRP. Considering this study was not originally conducted to assess ED, and ED was only evaluated by a single retrospectively ascertained question, thereby leading to a recall bias and the underestimation of true effect.

In particular, biomarker assistance in the diagnosis depended on their diagnostic performance. Previous studies had tested some markers and mediators of subclinical inflammation as candidates for detecting ED^5^, but most of these markers lacked specific and seemed to be unsuitable for routine clinical application. Few studies have explored the application of hs-CRP for improving the diagnosis of ED. Our results showed slightly poor performance compared with findings from Vlachopoulos *et al*.^27^ and Yao F *et al*.^16^, which found a similar diagnosis value of hs-CRP in diagnosing ED (AUC was 0.66 and 0.645, respectively.) Additionally, our ROC curve also demonstrated that serum hs-CRP had a moderate performance for differentiating moderate to severe ED and had relatively good performance for differentiating severe ED. Therefore, examining serum hs-CRP levels may provided meaningful predictive value for differentiating ED, although the diagnostic value were not ideal identified by the measurement of hs-CRP alone. In the future, it may be of interest to elucidate whether incorporating hs-CRP with other biomarkers or standard risk factors has a superior performance.

Understanding which exogenous exposures and endogenous biomarkers are causally involved in disease is a prerequisite for evaluating the efficacy of treatment. In this regard, clarifying inflammatory processes on ED provides a rationale for developing new treatment strategies that minimize the likelihood of causing sexual dysfunction. Recently, some studies indicated that pharmacotherapeutic regimens or behavioral modifications alleviated the inflammatory state and improved the erectile function. For example, a crossover trial involving 27 men with Vasculogenic ΕD found that the acute effect of sildenafil administration markedly decrease the hs-CRP level^31^. Similarly, Aversa and co-workers^32^ reported that chronic tadalafil administration also produced a concomitant decrease in CRP (−35% vs −8%, *p* < 0.05) and other markers of endothelial function. In addition, Lamina *et al*.^14^ showed that in hypertension men with ED, a continuous training programme as an effective nonpharmacological management improved erectile function and lowered CRP levels.

Moreover, the current measurement of hs-CRP assay can detect a much lower range and could expand the indications assessing the potential risk for ED in otherwise healthy individuals. Hopefully, utilizing serum hs-CRP levels as an adjunct to the well-known risk factors may provide a more comprehensive view of the ΕD patient’s overall risk profile and could aid in baseline screening, clinical settings and primary prevention of ED. However, scientific support for this proposal remains limited and the mechanisms underlying the associations between hs-CRP and ED have not been fully been elucidated. Several explanations can be proposed: considerable evidence supports that CRP impairs endothelial vasoreactivity via inhibiting endothelial nitric oxide synthase (eNOS) activity *in vitro* or interacts with endothelial cells through increasing the expression of P-selectin, E-selectin and vascular cell adhesion molecule-1 (VCAM-1) in cultured human endothelial cells^10,33–35^. Hence, all these profoundly down regulated the production of NO, thereby reducing maximal vasodilatation and blood flow and negatively influencing erectile function. More recently, a study from Chinese men reported that higher CRP concentration was associated with lower levels of total and free testosterone^36^, which indirectly provides available evidence supporting the association between hs-CRP levels and ED. Taken together, these findings suggest that hs-CRP may be involved in the genesis of endothelial dysfunction and play an important role in the pathogenesis of ED. However, more comprehensive laboratory studies involving the precise molecular mechanisms of hs-CRP and ED are needed.

Some limitations also merit consideration. Firstly, the cross-sectional design of our study could not identify the casual relationship. Secondly, although adequate control of confounding risk factors for ED had performed in our regression analyses, we still cannot exclude the unknown or unmeasured confounding variables. Third, our present study did not distinguish between vasculogenic and psychologic ED, which is subject to bias the result. Yet we have attempted to minimize confounding by excluded potential confounders. Finally, the results of our study in specific regions might not be generally applicable in other countries and ethnics. The strengths of our study include a large population-based sample (a broad age range) and relatively standard and structured questionnaires, which may limit the selection bias and strengthen the credibility and reliability of our results.

In summary, our study indicates that increased serum hs-CRP levels are associated with the severity of ED and an increased ED risk in the Chinese men. These findings suggest that hs-CRP may be of value as an inflammatory marker for the assessment of ED risk and play a critical role in the etiology of ED. Therefore, monitoring of hs-CRP is considered optimal in aiding ED risk assessment, pharmaceutical interventions and baseline screening.
